# Are There Familial Patterns of Symptom Dimensions in Obsessive-Compulsive Disorder?

**DOI:** 10.3389/fpsyt.2021.651196

**Published:** 2021-04-20

**Authors:** Srinivas Balachander, Sandra Meier, Manuel Matthiesen, Furkhan Ali, Anand Jose Kannampuzha, Mahashweta Bhattacharya, Ravi Kumar Nadella, Vanteemar S. Sreeraj, Dhruva Ithal, Bharath Holla, Janardhanan C. Narayanaswamy, Shyam Sundar Arumugham, Sanjeev Jain, YC Janardhan Reddy, Biju Viswanath

**Affiliations:** ^1^Obsessive-Compulsive Disorder Clinic, Department of Psychiatry, National Institute of Mental Health & Neuro Sciences, Bangalore, India; ^2^Accelerator Program for Discovery in Brain Disorders Using Stem Cells, National Institute of Mental Health & Neuro Sciences (NIMHANS), Bangalore, India; ^3^Department of Psychiatry, Dalhousie University, Halifax, NS, Canada; ^4^Department of Psychiatry Psychosomatics and Psychotherapy, University of Wuerzburg, Wuerzburg, Germany; ^5^Department of Neuroscience, Karolinska Institutet, Stockholm, Sweden; ^6^Department of Biomedicine, Aarhus University, Aarhus, Denmark

**Keywords:** OCD, obsessive-compulsive, familial, heritability, symptomatology, symptom dimensions, dimensions

## Abstract

**Background:** Obsessive-compulsive disorder (OCD) is a heterogeneous illness, and emerging evidence suggests that different symptom dimensions may have distinct underlying neurobiological mechanisms. We aimed to look for familial patterns in the occurrence of these symptom dimensions in a sample of families with at least two individuals affected with OCD.

**Methods:** Data from 153 families (total number of individuals diagnosed with DSM-5 OCD = 330) recruited as part of the Accelerator Program for Discovery in Brain Disorders using Stem Cells (ADBS) was used for the current analysis. Multidimensional Item Response Theory (IRT) was used to extract dimensional scores from the Yale-Brown Obsessive-Compulsive Scale (YBOCS) checklist data. Using linear mixed-effects regression models, intra-class correlation coefficients (ICC), for each symptom dimension, and within each relationship type were estimated.

**Results:** IRT yielded a four-factor solution with Factor 1 (Sexual/Religious/Aggressive), Factor 2 (Doubts/Checking), Factor 3 (Symmetry/Arranging), and Factor 4 (Contamination/Washing). All except for Factor 1 were found to have significant ICCs, highest for Factor 3 (0.41) followed by Factor 4 (0.29) and then Factor 2 (0.27). Sex-concordant dyads were found to have higher ICC values than discordant ones, for all the symptom dimensions. No major differences in the ICC values between parent-offspring and sib-pairs were seen.

**Conclusions:** Our findings indicate that there is a high concordance of OCD symptom dimensions within multiplex families. Symptom dimensions of OCD might thus have significant heritability. In view of this, future genetic and neurobiological studies in OCD should include symptom dimensions as a key parameter in their analyses.

## Introduction

Obsessive-compulsive disorder (OCD) is a complex neuropsychiatric illness, with a prevalence of 2–3% in the general population (1). Controlled family studies have identified an elevated risk of OCD in first-degree relatives of around 23% (2, 3), with odds ratios ranging from 11 to 32. Twin studies have also found heritability estimates of OCD to be around 30–60% (4), with higher heritability in pediatric OCD samples. Gene discovery efforts for OCD, especially those using genome-wide approaches have, however, yielded few consistent markers (5). Inability to replicate findings, in genetic and neurobiological research, is commonly attributed to the heterogeneity in the phenotypic presentation of OCD (6). To tackle this heterogeneity, several approaches have been employed to subtype the illness. These include using the age at onset (7–9), degree of insight (10–12), comorbidity profile [e.g. tic disorder (13, 14), depression/anxiety (15–17)], and familiality (18–20). One important approach in this direction has been that of OCD symptom dimensions.

Several factor analytic studies on OCD symptomatology have confirmed the existence of 5 factors (or dimensions, used interchangeably), which are contamination/washing, doubts/checking, symmetry/arranging, unacceptable/taboo thoughts (aggressive, sexual, religious) and hoarding (21, 22). Certain symptom dimensions are found to have specific clinical correlates, for e.g. symmetry/arranging is associated with earlier age at onset & family history (19, 23), greater comorbid depression & anxiety in those with forbidden thoughts (17, 24). Owing to major differences in neurobiology (25), treatment response (26) and other clinical features of patients with hoarding, it is now considered a separate diagnosis (27). Research on how the other symptom dimensions may differ from each other with respect to familial aggregation, genetics, or neurobiology, is still in its early stages (28).

Several studies have examined the familiality of broadly-defined OCD & clinical correlates of the familial form of OCD, but only a few of them have examined the familiality of individual symptom dimensions. Table 1 summarizes these studies. The largest of these studies (31) done in clinical populations analyzed the sample from the Obsessive-Compulsive Collaborative Genetics Study (OCGS), found significant co-occurrence between siblings, of contamination and hoarding dimensions. They also found that gender could play a role in the degree of sharing between the sibling pairs (35). Also reported similar findings with respect to contamination and hoarding dimensions (35). However, the ascertainment of information regarding OC symptoms in relatives was done only through administering a family history screen to the probands. A few other studies have found high concordance particularly for contamination symptoms (30, 33). Two twin studies have shown conflicting results regarding the commonality, i.e. shared vs. specific heritability of symptom dimensions. The smaller of the two studies (33) found commonality between all dimensions with specific heritability for contamination. However, the study done in the *TwinsUK* sample (34), in a much larger sample found that the best-fit model was one that included common and unique genetic/environmental factors for the symptom dimensions, and hoarding was found to have the lowest loading on the common factor.

**Table 1:** Studies that have examined familial sharing of symptom dimensions in Obsessive Compulsive Disorder (OCD).

**Study**	**Sample N**	**Ascertainment & Assessment**	**Statistical method**	**Findings**	**Limitations**
Leckman et al., 2003 (29)	128 siblings of Tourette Syndrome with OC symptoms (OCD in 45 of them), from 54 families with parents	Tourette Syndrome Association International Consortium, YBOCS applied on all recruited	Complex segregation analysis factor analysis-derived symptom dimensions	Aggressive/sexual/religious and symmetry/ordering had greater concordance among siblings, higher correlation between mother-child pairs	Only comorbid OCD/OCS in Tourette syndrome were studied
Chacon et al., 2007 (30)	40 siblings affected with OCD, from 18 families	Direct interview with all subjects, YBOCS checklist applied	ICC of factor analysis-derived symptom dimension scores	Greater concordance of contamination in male pairs, greater hoarding in female pairs	Small sample size, only sibling pairs examined
Hasler et al., 2007 (31)	418 subjects, comprised 173 pairs, 20 trios, 3 quartets from OCCGS	Direct interview with all subjects, YBOCS checklist applied	ICC of factor analysis derived symptom dimension scores	Significant ICCs for all factors, but very low values (Maximum ICC found for hoarding – 0.21) with gender dependence	Only sib-pairs, only early onset taken (mean age at onset = 8.7 years)
Pinto et al., 2008 (32)	OCCGS sample, 145 independent sibling pairs	Direct interview with all subjects, YBOCS checklist applied	ICC of item- & category-level factor analysis derived symptom dimension scores	Significant ICCs for hoarding, taboo thoughts, doubts/checking & contamination/cleaning. Symmetry/ordering not found significant	Same as above; also excluded tics & several other comorbidities
van Grootheest et al., 2008 (33)	331 monozygotic, 173 dizygotic female pairs from Virginia Twin Registry	Padua Inventory (Self-report)	Structural equation modeling of factor analyzed symptom dimensions	Common factor model for all dimensions had best fit, only contamination showed distinct genetic influence from other dimensions	Non-clinical sample, only OCS (not OCD) was evaluated, females only
Iervolino et al., 2011 (34)	4355 females from the *TwinsUK* Registry	Obsessive Compulsive Inventory- Revised (self-report)	Multivariate Twin modeling	Common pathway model did not fit, independent genetic & shared environmental	Non-clinical sample, only OCS, only female twin pairs
Brakoulias et al., 2016 (35)	121 OCD probands with family history of OC symptoms	Probands assessed with V-OCI, Symptoms in family members derived from Family history screen administered on probands	*t*-tests comparing those with FDR having a particular dimension vs. those without	High sharing of contamination & hoarding, low for all other dimensions	Relatives not interviewed
Chacon et al., 2018 (36)	66 children of OCD probands	Children screened for OCS using a 5-question screen, YBOCS applied on parent probands only	Comparison of YBOCS checklist of parents of children with vs. without OCS	Children with OCS more commonly had probands with contamination/washing	Symptom sharing not analyzed
Burton et al., 2018 (37)	16,718 youth (general population)	Toronto Obsessive-Compulsive Scale	Univariate & multivariate latent trait & twin modeling	Hoarding had the highest unique heritability, all other factors also had specific	Non-clinical sample

*OCS, obsessive-compulsive symptoms; OCCGS, Obsessive-Compulsive Consortium for Genetic Studies; YBOCS, Yale-Brown Obsessive-Compulsive Scale; ICC, Intraclass Correlation Coefficient; V-OCI, Vancouver Obsessive Compulsive Inventory*.

Overall, the studies have shown heterogeneous findings, which might result from the varying methodology. For example, some of the studies have focused primarily on a particular phenotype, such as comorbid Tourette syndrome, early-onset symptoms, female subjects etc., which may limit the generalizability of the results. Some studies have been conducted on non-clinical analog populations. Other methodological issues include varying methods of clinical assessment and type of relationships with probands studied (some studies have focused on sibling/twin pairs alone).

Hence, from the available research, it is still difficult to conclude whether the individual symptom dimensions in OCD are heritable, or at least have a familial concordance. This is important to study, especially in clinical populations, as familiality is one of the criteria originally proposed by Robins & Guze (38), to establish the validity of a construct. Additionally, there are no studies on the effect of specific relationships, like sex-concordance and parent-of-origin (i.e. imprinting) in the transmission of the OC symptom dimensions.

The aim of this current study was to examine the familial patterns in the co-aggregation of these specific symptom dimensions in a sample of families with multiple first-degree relatives affected with OCD. We hypothesized that all symptom dimensions would show familial concordance and that the degree of concordance may differ based on gender and type of relationship between the affected individuals.

## Materials and Methods

### Clinical Recruitment

We screened all individuals seeking treatment for OCD at the speciality OCD Clinic of the National Institute of Mental Health and Neurosciences (NIMHANS), Bangalore between July 2016 and December 2019 for the presence of OCD in their first-degree relatives. Individuals were asked about a family history of OCD for the purpose of recruitment into the Accelerator Program for Discovery in Brain Disorders using Stem Cells (ADBS) (39). The study is approved by the Institute Ethics Committee and all participants gave written informed consent to participate in the study.

Out of a total of 1,354 subjects with OCD, 330 (24%) individuals, belonging to 153 families were found to have familial OCD (that is having a first-degree relative, either a parent or a sibling, with OCD). A diagnosis of OCD was ascertained first by interviewing at least three family members, for a family history of OCD and then confirmed later by directly interviewing the affected family members by asking questions from the OCD section of the MINI International Neuropsychiatric Interview (MINI) 7.0.0 (40).

### Assessments

All subjects underwent a detailed clinical assessment using the Mini International Neuropsychiatric Interview (MINI) 7.0.0 (40) and the Yale-Brown Obsessive Compulsive Scale (Y-BOCS) symptom checklist and the severity measure (41, 42). The diagnosis of OCD was confirmed by two clinicians, at least one being a consultant psychiatrist specialized in the diagnosis of OCD. All raters underwent training with inter-rater reliability exercises for the Y-BOCS every 3 months using interview transcripts, which yielded high reliability indices for the total score (Cronbach's alpha = 0.83–0.89), and for all the main symptom categories in the checklist (Cohen's kappa = 0.90–0.96).

### Statistical Analysis

#### Sample Size and *post-hoc* Statistical Power Estimation

Sample size estimation & *post-hoc* power analysis was carried out (43) using the package ICC Sample Size (44). With the given sample size of 153 families, the minimum ICC value which can be reliably detected with a statistical power of 0.8 is 0.20. As we intend to also look at pairs of specific relationship types within the sample, we extrapolated this power analysis for various sample sizes and ICC estimates, as shown in Supplementary Figure 1. The ICC value increases to 0.24 at *N* = 100 and to 0.34 at *N* = 50.

#### Item Response Theory Analysis

The Item response theory (IRT) has gained popularity as a method to identify latent traits or dimensions within categorical/binary data. It is known to have several advantages over approaches based on classical test theory, such as factor analysis. IRT involves the estimation of certain parameters that helps in understanding the relationship between each item in the scale and the latent trait/dimension(s) that we aim to measure. One of the most commonly used IRT methods is the 2-parameter logistic (2-PL) model, wherein each scale item is gauged based on a “discrimination” parameter and a “difficulty” parameter. The discrimination parameter indicates the degree of specificity of that item that latent trait, and the difficulty parameter indicates the likelihood (or ‘ability’) of a subject endorsing the item. These are represented graphically as item response characteristics curves, with difficulty indicated in the x-axis and discrimination in the y-axis, respectively. Hence, the identification of latent traits/dimensions and their scores, are considered to have greater accuracy with IRT than with the other methods (45).

Using the irt.fa function in the “psych” package in R (46), multidimensional item response theory analysis (MIRT) with the 2-parameter logistic (2-PL) model was carried out, with the main categories of the Y-BOCS checklist items. From the “Miscellaneous” categories of the obsession and compulsion checklists, only those items which were present in more than 10% of the sample were included. As part of the MIRT, exploratory factor analysis was done using the “generalized least squares” method, from a tetrachoric correlation matrix of the Y-BOCS symptom checklist items. An orthogonal rotation using the “varimax” method was employed. The resultant loadings from the factor analysis are transformed to item discrimination parameters. The “tau” parameter from the tetrachoric correlations, combined with the item factor loading are then used to estimate item difficulties. As the number of factors to be extracted can be pre-specified, we ran the same analysis starting from 2-factor up to a 6-factor model. We compared the fit indices (Bayesian Information Criteria, Comparative Fit Index & Root Mean Square of Approximation) of each of these models. The final model was chosen after considering both the fit indices as well as concordance with the existing literature on symptom dimensions from the factor-analytic studies on OCD (47). Using the parameter estimates for discrimination and difficulty IRT-based scores were derived for each individual subject, to take up for the familiality analysis.

#### Familiality Analysis

Using the “lme4” (48) and “performance” (49) packages in R, we used a linear mixed-effects model to compute the intra-class correlation coefficient (ICC) values for each symptom dimension. The ICC has been used in several other studies (31) to measure the level of sharing of phenotypic traits between family members, and was originally developed for this purpose (50).

Sex and age at onset were included as fixed-effect covariates, in order to regress out their influences on phenotypic expression. Several previous studies have indicated that symptom dimensions vary based on sex (51–53) and age at onset of illness (9, 54). The “Family ID” was included as a random-effects variable, and the ICC was calculated as the ratio of the residual variance between families (or pairs) to the total variance between all subjects (55). We report “adjusted-ICC” values in the output, due to the non-Gaussian distribution of the residuals (56). Similar analyses were carried out separately to estimate ICCs for each type of relationship (e.g. parent-offspring, sibling-sibling, sex-concordant and sex-discordant). Sex was not added as a covariate while analyzing the gender-concordant pairs, but the age at onset was included in all of them. In order to estimate standard errors and 95% confidence intervals for the ICCs, a bootstrapping procedure, run for 10,000 iterations, was employed for each of the mixed-effect models.

## Results

### Sample Characteristics

**Figure 1:**
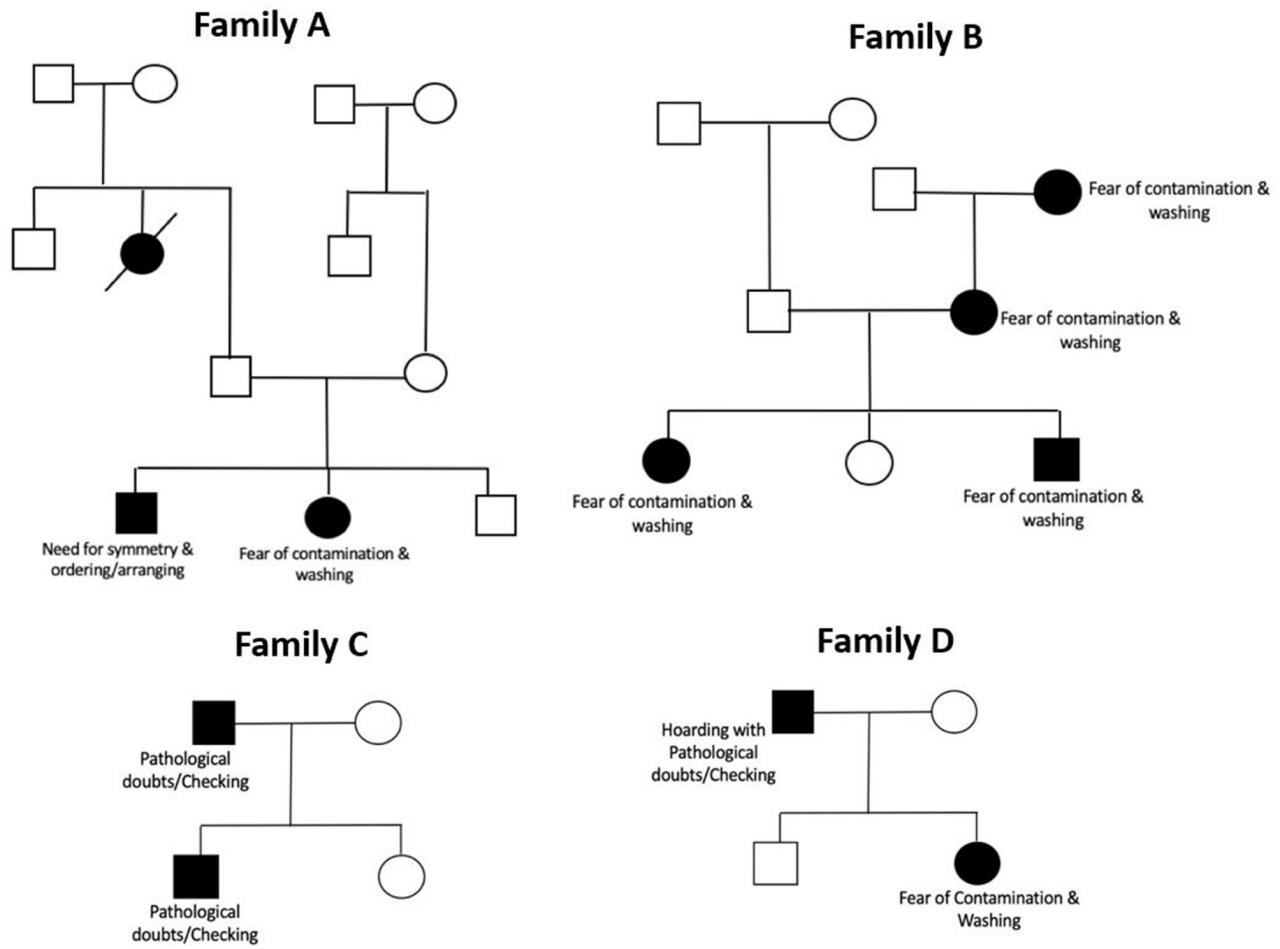
Representative pedigrees from the sample showing principal symptoms in affected member. The pedigrees have been illustrated using standardized pedigree nomenclature (57). Boxes represent male sex, and circles represent female sex. Black shading within a box/circle indicates the disease affectation status (OCD), while an unshaded box/circle indicates that the individual is unaffected. Diagonal line through a square/circle indicates deceased status.

Figure 1 shows four representative pedigrees from our sample, along with the principal symptom dimension of the affected individuals in the family. The sample consisted of 132 families with two affected members, 19 families with three affected members, one family with three affected members and one family with five affected members. There were no families with concordant twins (monozygotic or dizygotic) in the sample.

Table 2 shows the clinical and sociodemographic details of the total sample. Juvenile-onset OCD (age at onset before 18 years) was seen in 112 (34%) of the sample. Supplementary Figures 1, 2 also show the differences in the age at onset of OCD by generation, and be sex. Additionally, a majority of the sample (84%) had at least one lifetime comorbidity, as assessed using the MINI.

**Table 2:** Clinical characteristics of the sample (*N* = 330).

**Characteristic**		***n* (%) or Mean (SD)**
Sex	Male	166 (50.5%)
	Female	164 (49.5%)
Age at assessment (years)		36.15 (14.24)
Age at onset (years)		21.84 (8.58)
YBOCS Severity Rating	Obsession Sub-total	11.75 (3.70)
	Compulsions sub-total	11.09 (4.26)
	Total	22.6 (7.70)
	Insight (Item-11)	1.29 (0.74)
	Avoidance	1.39 (0.95)
CGI-S		3.91 (1.20)
Poor Insight (YBOCS-11 “3” or “4”)		27 (9.1 %)
YBOCS Checklist Items (Lifetime)		
Obsessions	Contamination	214 (64.8%)
	Somatic	31 (9.4%)
	Aggressive	97 (29.4%)
	Sexual	62 (18.8%)
	Religious	87 (26.4%)
	Hoarding	45 (13.6%)
	Pathological Doubts	175 (53%)
	Need for Symmetry	112 (33.9%)
Compulsions	Washing	220 (66.7%)
	Checking	184 (55.8%)
	Repeating	115 (34.8%)
	Counting	24 (7.3%)
	Arranging/Ordering	105 (31.8%)
	Collecting	36 (10.9%)
	Mental Compulsions	141 (42.7%)
Comorbidity (Lifetime)		
Major Depressive Disorder		148 (44.9%)
Dysthymia		36 (10.9%)
Hypo/Mania		22 (6.7%)
Generalized Anxiety Disorder		48 (14.5%)
Panic disorder		24 (7.31%)
Agoraphobia		17 (5.2%)
Social Anxiety Disorder		22 (6.7%)
Psychosis		19 (5.8%)
Attention Deficit Hyperactivity Disorder		9 (2.7%)
Substance Use Disorder (Any - excluding Nicotine)		9 (2.7%)
Tic Disorder		19 (5.8%)

### Item Response Theory Analysis

**Figure 2:**
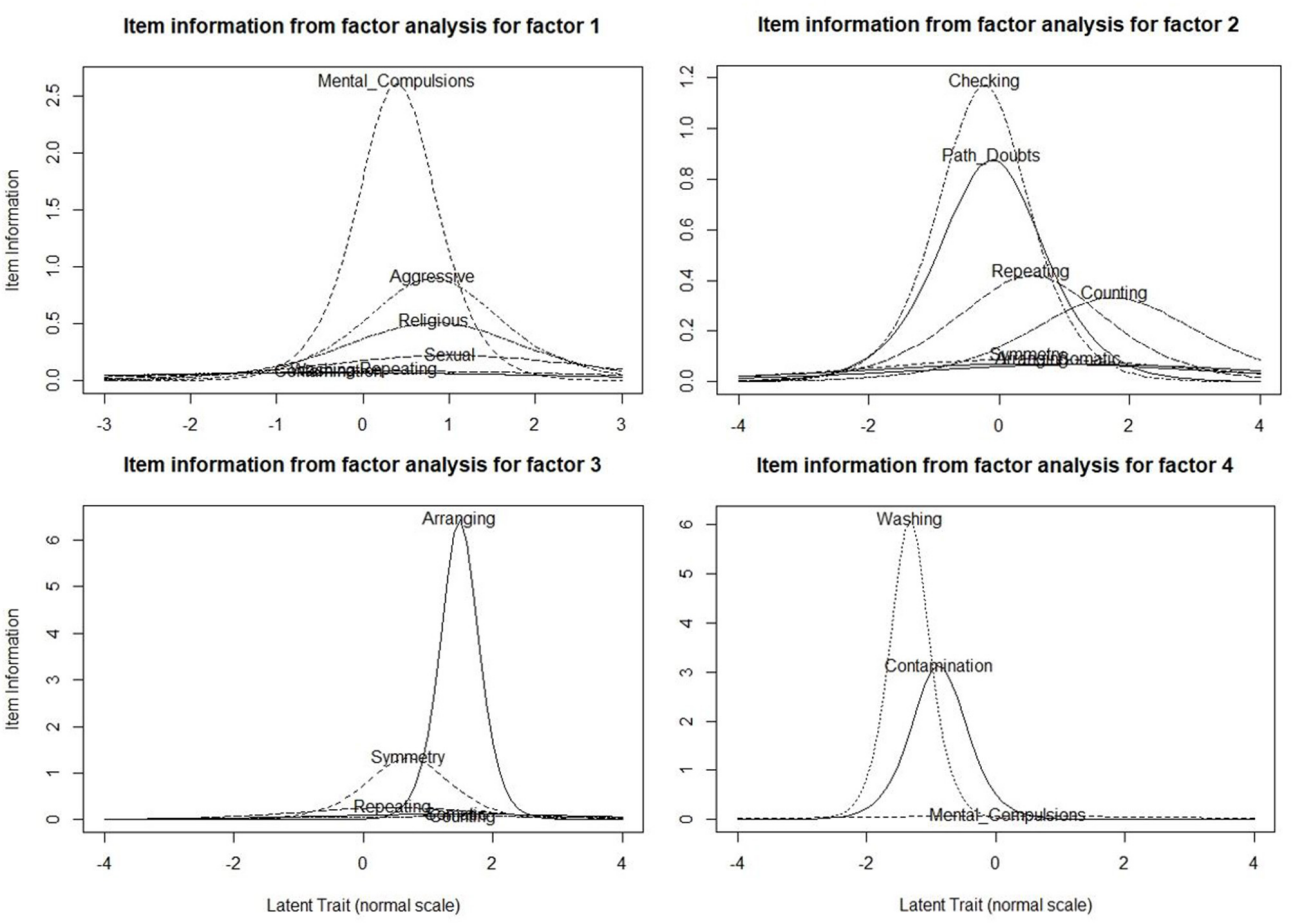
Item Information Curve (IIC) Plots of the Multi-dimensional Item Response Theory analysis done with the Yale-Brown Obsessive-Compulsive Checklist Items (*N* = 330). These plots represent the item information curves (IICs) for the items that are loaded within each factor. The x-axes represent the “difficulty” parameter (lesser “difficulty” means greater likelihood of the subject endorsing this item), and the y-axes represent the “discrimination” parameter. IICs with high peaks and relatively narrow spread indicate high discrimination, or high specificity of the item for that particular factor.

The results of the item-response theory analysis are shown in Figure 2. As shown in the figure, the four factors were as follows: Factor 1 included mental compulsions along with sexual, religious and aggressive obsessions, Factor 2 included pathological doubts with checking, repeating and counting compulsions, Factor 3 included need for symmetry obsessions along with ordering/arranging compulsions, Factor 4 was fear of contamination with cleaning/washing compulsions. The YBOCS checklist item of “somatic” obsessions did not appear to have significant loading with any of the factors. This model was found to have the following fit indices: the cumulative variance explained by the factor analysis step was 68%, the comparative fit index was 0.95, and the root mean square error of approximation was 0.076 (90% CI 0.067–0.085), all of which indicate an acceptable level fit for the model.

### Mixed-Effects Intraclass Correlation Co-efficient Analyses

**Figure 3:**
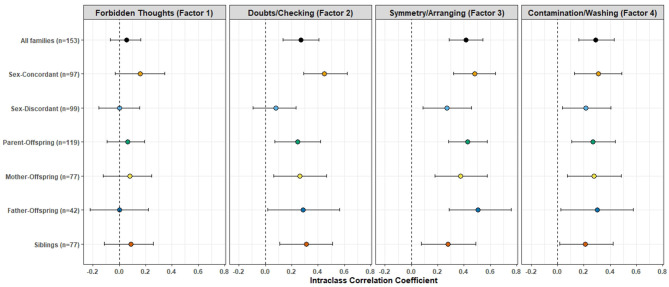
Intra-class Correlation Coefficients (ICC) of the factor analysis-derived symptom dimension scores between first-degree relative pairs. The colored dots represent the ICC value, the error bars represent their 95% confidence intervals, for each symptom dimension and across different relationship types. See Supplementary Table 1 for the source data.

Figure 3 shows the results of the ICC values derived from the mixed-effect modeling, for the overall sample and for each specific relationship type (see Supplementary Table 1 for the actual ICC values). Only Factor 2 (Doubts/Checking), Factor 3 (Symmetry/Arranging) and Factor 4 (Contamination/Washing) were found to have significant ICC values when all members within families were included, regardless of the gender or type of relationship. The highest ICC was seen for symmetry/ordering (0.41), followed by contamination/washing (0.29) and then in pathological doubts/checking (0.27) dimension. The ICC values in the sex-concordant pairs were higher than those in the sex-discordant pairs for every factor. The ICC values and their 95% confidence intervals do not appear to deviate markedly from each other when parent-offspring and sibling pairs were looked into specifically. Significant ICC values were found in every relationship type for symmetry/ordering and contamination/washing dimension. ICC values were not found to be significant in any of the specific relationship types for Factor 1 (“Forbidden thoughts”), and in the gender-discordant pairs for doubts/checking dimension. We also conducted *post-hoc* analyses on a subset of families having multiple members (≥2) having OCD with comorbid depression (either Major Depressive Disorder or Dysthymia). We found similar results even in this subset; significant ICC values were found for all factors except Factor 1 (“forbidden thoughts”) (Supplementary Table 2).

## Discussion

To our knowledge, this is the first study to report familial aggregation of symptom dimensions among first-degree relatives affected with OCD in a large sample of multiplex OCD families. The study also analyzed how the sharing of symptom dimensions might be influenced by the type of relationships between the affected members, and gender.

We originally hypothesized that all symptom dimensions would show strong familial concordance. The main finding of our study showed that only three of the symptom dimensions, which include “symmetry/arranging,” “contamination/washing” and “doubts/checking” had significant familial concordance. The “forbidden thoughts” dimension, which includes aggressive, sexual and religious obsessions along with mental compulsions, did not show significant concordance. Also, higher degrees of concordance for all symptom dimensions was found when the affected members within a family were of the same sex, in contrast to when they were of the opposite sex. There were no major differences between parent-offspring pairs (both mother-offspring as well as father-offspring) and sibling pairs.

Similar findings of high familial concordance for the contamination/washing dimensions have been demonstrated in two previous studies (31, 35). A previous twin study that analyzed the sample from the Virginia Twin Cohort (33) found contamination to have a distinct genetic heritability, all other dimensions were better explained by a latent common factor model. In contrast, a subsequent study from the *TwinsUK* registry with a much larger sample size found only the hoarding dimension to have distinct genetic influences (34). However, as these studies were done in non-clinical populations, it is not clear how these self-reported “OC-like” behaviors may differ from symptoms in OCD. The heritability of the contamination/washing dimension hence needs to be examined further.

Studies that have compared familial and sporadic OCD have indicated the high occurrence of symmetry/arranging dimension in familial OCD samples (18, 23, 58). This was also shown when comparing early-onset OCD to adult-onset and tic-related OCD to non-tic related OCD, showing higher rates of symmetry/arranging (14). A recent candidate gene study from our center, evaluating a polymorphism in the DRD4 gene found a specific association with the symmetry/arranging dimension (59). All of these indicate that there may be a higher genetic contribution associated with this factor.

The “forbidden thoughts” factor was found to have the least degree of familial concordance, even after accounting for comorbid depression. The low familiality of this dimension, especially between siblings, is in contrast with the findings of the OCD Collaborative Genetics Study (OCGS) (31), which reported the highest concordance for this factor among sibling pairs. Their sample consisted of early-onset OCD with predominantly females (70%), and were Caucasians. It may hence be important to examine this separately in early and late onset cohorts, and further across different ethnicities as well.

Previous factor analysis studies in OCD have shown discrepant findings with respect to aggressive/harm & checking- related symptoms. While some studies have shown checking compulsions to load with aggressive obsessions (60, 61), many others (32, 62, 63) including several from our center (12, 64, 65) found doubts & checking to load separately from aggressive obsessions (which loads with forbidden/taboo thoughts). In the current study “doubts” were coded separately from aggressive obsessions, which could have resulted in a factor structure different from the OCGS study. This could have thus influenced the findings with respect to the familiality of the “forbidden thoughts” dimension.

### Strengths of the Study

There are several strengths to this study. First, the study is unique in that the sample included multiplex OCD families of OCD which is different from the previous studies that have looked at only sibling pairs or one study which looked only at parent-offspring pairs. This helped in examining the patterns between specific relationship types in the sample. All participants were evaluated by interviewing them directly, and the assessments were carried out by trained raters with high inter-rater reliability. This is a key advantage over several of the studies, which used self-report tools or assessed only one of the subjects within the family (see Table 1 for details).

The sample was ascertained from a tertiary-care speciality OCD clinic, and information was collected about all first and second-degree relatives in the families. Despite this, there was an uneven sex distribution in the parental generation. Although the overall sex ratio was even (nearly 1:1, as shown in Table 2), in the parent-child pairs, the ratio of number of female: male parents was 77:42 (as depicted in Figure 3). One might also speculate that there may be a “cohort effect.” That is, the males in the older generation who had the phenotype of familial OCD with an earlier age at onset of symptoms, greater comorbidities and possibly poorer overall outcome, may have had lesser fecundity and hence were poorly represented in our sample. Females, on the other hand, have a later onset of OCD, and are also commonly known to have onset of OCD after the first child-birth (66). This phenomenon, of a “cohort effect” has been reported previously in longitudinal cohort studies of schizophrenia (67).

### Limitations

Despite the relatively large overall sample size (330 subjects from 153 families), the power analysis indicated that the minimum ICC that could be reliably estimated was 0.2 with the total sample, and this increased gradually for smaller sample size. Hence, the results of the sub-analyses done for the specific relationship pairs need to be interpreted with caution.

Another limitation of our study was the use of a checklist for assessing OCD symptoms, which is categorical/dichotomous measure, hence the factor scores that were derived for each subject may not indicate a true “severity” of that particular dimension for the subject. This could have been overcome by the use of the dimensional YBOCS (D-YBOCS), which gives a separate severity score ranging from 0 to 15 across each symptom dimension (68). However, the D-YBOCS is used only as a cross-sectional measure and its reliability in measuring the lifetime severity of these symptom dimensions is uncertain. Unaffected FDRs were not included in the analysis as very few of them reported symptoms that could be tapped by the YBOCS checklist. Possibly, the additional use of either the D-YBOCS or a self-reported measure like the Obsessive-Compulsive Inventory – Revised (OCI-R) (69) or the Padua Inventory (70) may have been more sensitive to pick up sub-threshold OC symptoms and symptoms with forbidden/taboo content.

In addition to the above limitation, it is difficult to draw inferences about genetic mechanisms such as imprinting/silencing due to confounding environmental/psychosocial influence. One might still argue that these could be behaviors that are “learned” or “taught” between family members. Family accommodation is one such factor that can play a significant role in the sharing of symptoms. Accommodation refers to responses of the patient's family (typically parents, spouse or even children) to his/her obsessive-compulsive symptoms, and includes behaviors such as directly participating in compulsions, or helping to avoid triggers of obsessions or distress (71). Investigating if such accommodative behaviors may have preceded the onset of OCD in the affected FDRs in multiplex OCD families would help understand this further. However, large-scale studies of OC symptoms in non-clinical twin samples have found that the sharing of symptoms between twin pairs is more likely to be due to genetic than environmental factors, with heritability estimates of around 60–100% (34, 37).

## Conclusions

We conclude that the symptom dimensions, particularly checking, washing & arranging have a robust familial basis. Efforts are being made to validate symptom dimensions by identifying each of their unique clinical and neurobiological correlates. High familiality of these specific symptom dimensions further emphasizes the need for such an approach, in order to deconstruct the complex phenotype of OCD. Stratifying patients into such homogeneous sub-groups based on symptom dimensions may substantially improve statistical power and facilitate discovery of reproducible genetic and imaging signatures of the illness. Further research into the clinical utility of these symptom dimensions, such as response to specific treatments is also warranted and likely to have an important role in developing “personalized” treatment options for OCD.

## Data Availability Statement

The raw data supporting the conclusions of this article will be made available by the authors, without undue reservation.

## Ethics Statement

The studies involving human participants were reviewed and approved by Institute Ethics Committee, National Institute of Mental Health&Neuro Sciences (NIMHANS), Bangalore. Written informed consent to participate in this study was provided by the participants' legal guardian/next of kin.

## Author Contributions

SB, FA, AK, MB, and DI were involved in the data collection for this study. SB, VS, RK, and BH were involved in the statistical analysis. SM and MM critically reviewed the manuscript and provided inputs to improve the analysis & interpretation of the study results. SB, SJ, BV, YR, JN, and SA contributed to study planning, conceptualization, and manuscript preparation & review. All authors contributed to the article and approved the submitted version.

## Conflict of Interest

The authors declare that the research was conducted in the absence of any commercial or financial relationships that could be construed as a potential conflict of interest.
