# Supplementary material for: Are There Familial Patterns of Symptom Dimensions in Obsessive-Compulsive Disorder?
Source: Front Psychiatry. 2021 Apr 20;12:651196. doi: 10.3389/fpsyt.2021.651196 (PMC8093508; doi:10.3389/fpsyt.2021.651196)
Supplement: Supplementary file 1 [file Data_Sheet_1.docx]

Supplementary Material

Are there familial patterns of symptom dimensions in obsessive-compulsive disorder?

**Supplementary Figure 1. Power analysis & Sample size estimation**


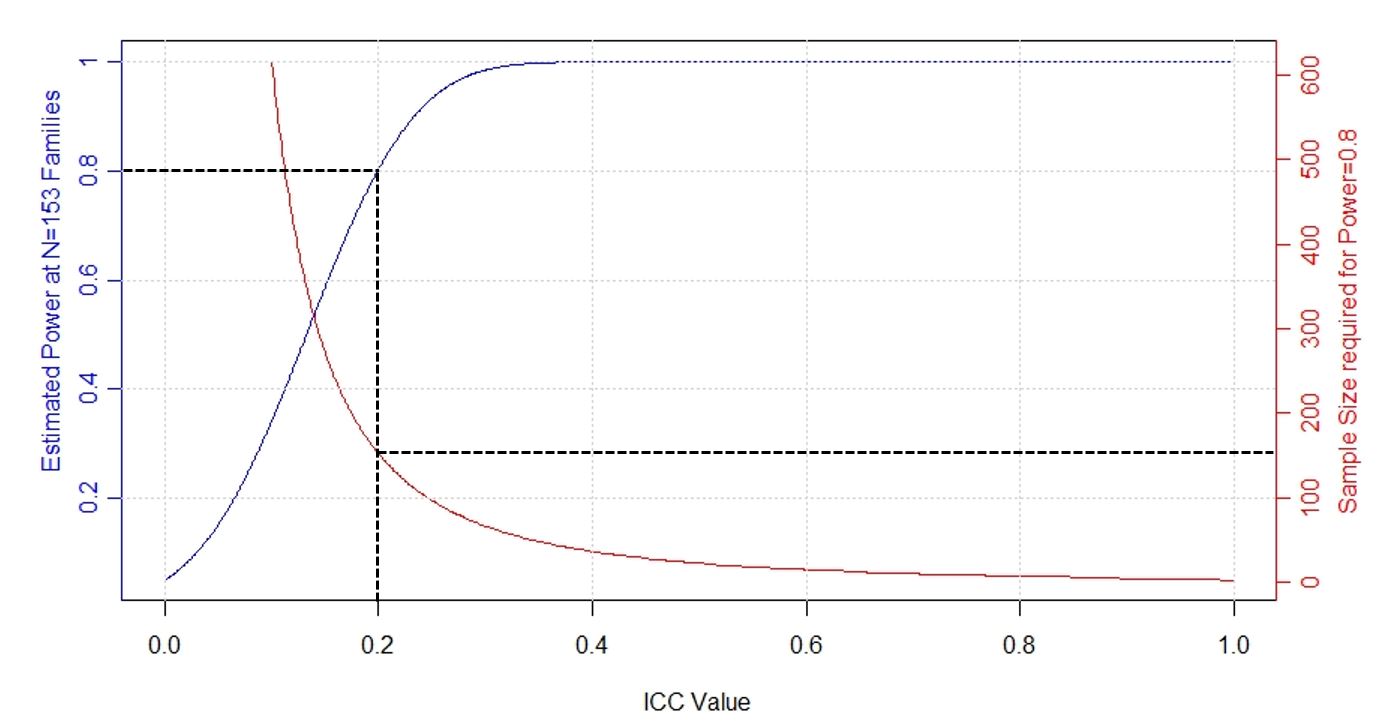


The blue line indicates the statistical power that was achieved with a sample size of 153 families, for each estimate of the intraclass correlation coefficient (ICC). The red line indicates the sample size that is required in order to achieve a statistical power of 0.8, for each estimated ICC value.

Results of the power analysis show that for the given sample size (number of families) of 153, the minimum ICC value which can be reliably detected with a statistical power of 0.8 is 0.20

**Supplementary Figure 2: Survival plots showing differential mean age at onset in OCD in parent-child pairs as compared to the sibling-sibling pairs**


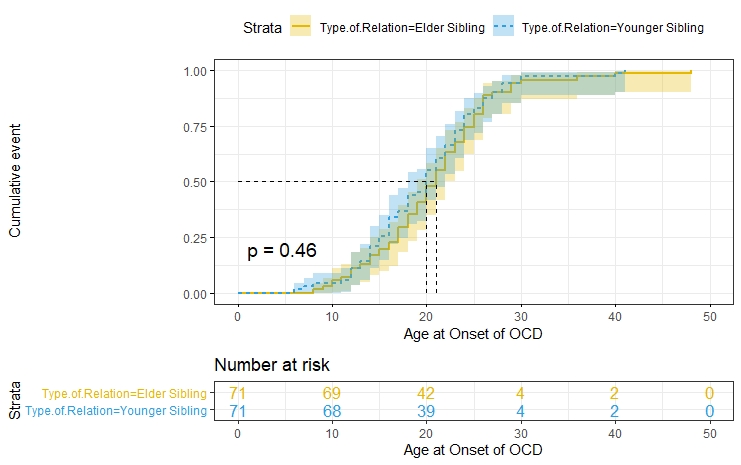

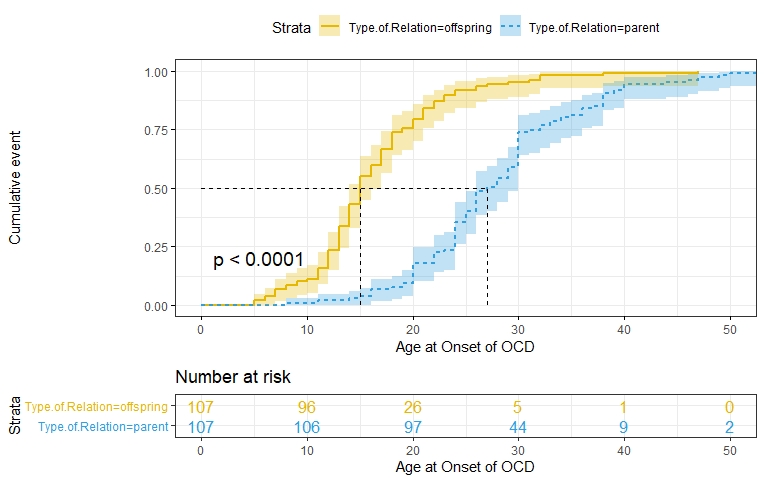


Figure in the left shows that in the parent-offspring pairs, the age at onset of the children were around 16 years, but those of parental generation was around 27 years. In the sibling pairs (grouped as elder vs younger sibling), the mean in both groups was around 20 years, and the difference was not statistically significant

**Supplementary Figure 3: Survival plots showing gender differences in mean age at onset in OCD**


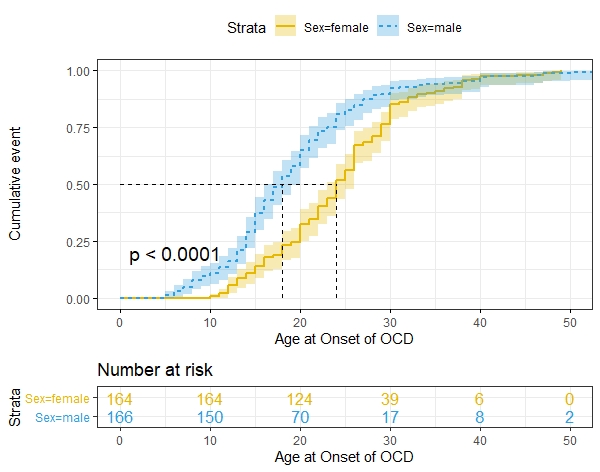


Males had an earlier mean age at onset at 19.5 years, females at around 24 years

**Supplementary Table 1: Intra-class Correlation Coefficients (ICC) of the factor analysis-derived symptom dimension scores between first-degree relative pairs (Data from which Fig 3 was derived)**

| **Relationship Type** | **Dimension** | **ICC** | **Lower-CI** | **Upper-CI** |
| --- | --- | --- | --- | --- |
| All families (n=153) | Forbidden Thoughts (Factor 1) | 0.057 | -0.067 | 0.165 |
| All families (n=153) | Doubts/Checking (Factor 2) | 0.269 | 0.135 | 0.405 |
| All families (n=153) | Symmetry/Arranging (Factor 3) | 0.414 | 0.286 | 0.543 |
| All families (n=153) | Contamination/Washing (Factor 4) | 0.290 | 0.160 | 0.432 |
| Parent-Offspring (n=119) | Forbidden Thoughts (Factor 1) | 0.064 | -0.093 | 0.192 |
| Parent-Offspring (n=119) | Doubts/Checking (Factor 2) | 0.245 | 0.072 | 0.420 |
| Parent-Offspring (n=119) | Symmetry/Arranging (Factor 3) | 0.428 | 0.282 | 0.575 |
| Parent-Offspring (n=119) | Contamination/Washing (Factor 4) | 0.268 | 0.110 | 0.437 |
| Siblings (n=77) | Forbidden Thoughts (Factor 1) | 0.089 | -0.112 | 0.257 |
| Siblings (n=77) | Doubts/Checking (Factor 2) | 0.310 | 0.111 | 0.510 |
| Siblings (n=77) | Symmetry/Arranging (Factor 3) | 0.277 | 0.077 | 0.488 |
| Siblings (n=77) | Contamination/Washing (Factor 4) | 0.211 | 0.016 | 0.423 |
| Sex-Concordant (n=97) | Forbidden Thoughts (Factor 1) | 0.158 | -0.033 | 0.344 |
| Sex-Concordant (n=97) | Doubts/Checking (Factor 2) | 0.449 | 0.290 | 0.623 |
| Sex-Concordant (n=97) | Symmetry/Arranging (Factor 3) | 0.480 | 0.321 | 0.640 |
| Sex-Concordant (n=97) | Contamination/Washing (Factor 4) | 0.309 | 0.128 | 0.487 |
| Sex-Discordant (n=99) | Forbidden Thoughts (Factor 1) | 0.000 | -0.154 | 0.154 |
| Sex-Discordant (n=99) | Doubts/Checking (Factor 2) | 0.080 | -0.093 | 0.232 |
| Sex-Discordant (n=99) | Symmetry/Arranging (Factor 3) | 0.269 | 0.087 | 0.456 |
| Sex-Discordant (n=99) | Contamination/Washing (Factor 4) | 0.215 | 0.036 | 0.407 |
| Mother-Offspring (n=77) | Forbidden Thoughts (Factor 1) | 0.079 | -0.123 | 0.245 |
| Mother-Offspring (n=77) | Doubts/Checking (Factor 2) | 0.260 | 0.064 | 0.463 |
| Mother-Offspring (n=77) | Symmetry/Arranging (Factor 3) | 0.373 | 0.180 | 0.575 |
| Mother-Offspring (n=77) | Contamination/Washing (Factor 4) | 0.277 | 0.076 | 0.484 |
| Father-Offspring (n=42) | Forbidden Thoughts (Factor 1) | 0.001 | -0.222 | 0.222 |
| Father-Offspring (n=42) | Doubts/Checking (Factor 2) | 0.288 | 0.019 | 0.564 |
| Father-Offspring (n=42) | Symmetry/Arranging (Factor 3) | 0.504 | 0.288 | 0.759 |
| Father-Offspring (n=42) | Contamination/Washing (Factor 4) | 0.301 | 0.025 | 0.574 |

**Supplementary Table 2: Intra-class Correlation Coefficients (ICC) of the factor analysis-derived symptom dimension scores between first-degree relatives having OCD and comorbid lifetime depression [N (individuals) = 107; N (families) = 47]**

| **Dimension** | **ICC** | **Lower-CI** | **Upper-CI** |
| --- | --- | --- | --- |
| Forbidden Thoughts (Factor 1) | 0.00 | -0.191 | 0.093 |
| Doubts/Checking (Factor 2) | 0.437 | 0.231 | 0.660 |
| Symmetry/Arranging (Factor 3) | 0.232 | 0.007 | 0.468 |
| Contamination/Washing (Factor 4) | 0.259 | 0.037 | 0.489 |
